# Comparison of the Incidence and Magnitude of Hyponatremia Among Patients With Poststroke Depression Receiving Either Escitalopram or Sertraline

**DOI:** 10.1002/prp2.70041

**Published:** 2024-12-02

**Authors:** Lina Naseralallah, Zahra Noureddine, Somaya Koryash

**Affiliations:** ^1^ Clinical Pharmacy Department Hamad Medical Corporation Doha Qatar

**Keywords:** adverse drug reactions, depression, hyponatremia, poststroke depression, selective serotonin reuptake inhibitors, stroke

## Abstract

Depression is the most frequent psychiatric condition experienced in stroke survivors. Selective serotonin reuptake inhibitors (SSRIs) are frequently used as first‐line antidepressants; however, they have been strongly associated with hyponatremia which, in poststroke patients, can worsen outcomes. This study aims to determine and compare the incidence and magnitude of hyponatremia and potential risk factors in patients receiving either escitalopram or sertraline for the management of poststroke depression (PSD). A retrospective observational study involving all hospitalized patients who received either escitalopram or sertraline for the treatment of PSD. Electronic medical records were reviewed over a 5‐year period with data collected on various demographic, laboratory, comorbidity, and medication‐related variables. Data were analyzed using multivariate logistic regression. A total of 401 patients met the inclusion criteria. Overall, 36.7% of patients experienced hyponatremia, with 67 (38.3%) cases in patients receiving escitalopram and 76 (33.6%) in sertraline group. The median drop in sodium level from baseline was 5 mmol/L in both groups; with the majority of cases being of mild nature (73.1% and 69.7% for escitalopram and sertraline, respectively). Findings from the multivariate logistic regression did not yield a model with significant association (*p* = 0.353). Escitalopram and sertraline were both associated with an increased risk of hyponatremia in poststroke patients, with most cases being mild. There was no significant difference between treatment arms regarding the incidence or magnitude of hyponatremia. Caution should be exercised when prescribing escitalopram or sertraline.

## Graphical abstract

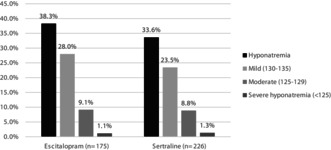

## 1 Introduction

A stroke is an acute neurologic injury that can be classified into two major types: ischemic and hemorrhagic [1]. An ischemic stroke occurs when blood supply to the brain is disrupted due to thrombosis, embolism, or systemic hypoperfusion, whereas a hemorrhagic stroke occurs when a weakened blood vessel ruptures and bleeds into the brain [2]. Globally, ischemic strokes account for 62%, while hemorrhagic strokes account for 38% [3]. One in four adults over the age of 25 will have a stroke in their lifetime and over half of those people will die as a result [3]. The literature shows that stroke incidence in Qatar has progressively increased over the years with incidence rising from 36.5/100 000 in 2014 to 48.4/100 000 in 2019 [4]. Another Qatar‐based study estimated 85.3% of stroke patients to have an ischemic stroke, whereas hemorrhagic stroke accounted for 14.7% over the span of 8 years (2014–2022) [5].

For stroke survivors, poststroke complications can significantly impact their physical, cognitive, emotional, and social functioning. Studies have shown that stroke survivors suffer a wide range of neuropsychiatric diseases such as depression, anxiety, and agitation [6, 7]. Depression is the most frequent psychiatric condition poststroke, occurring in approximately one‐third of stroke survivors at any one time, with the frequency highest in the first year [6, 7]. Depressed mood, apathy, weight changes, sleep disturbances, exhaustion, worthlessness, and anhedonia are the primary clinical signs of poststroke depression (PSD), with the first two being the most prominent [8]. Patients with PSD are at a higher risk for suboptimal recovery, recurrent vascular events, poor quality of life, and mortality, hence necessitating early detection and treatment [9, 10, 11]. Patients diagnosed with PSD should be considered for a trial of antidepressant medication to optimize rehabilitation efforts and enhance the recovery process. Although no one drug or drug class has been found to be superior for PSD treatment, side effect profiles suggest that selective serotonin reuptake inhibitors (SSRIs) may be favored in this patient population [12]. SSRIs are frequently used as first‐line antidepressants because of their efficacy, tolerability, and safety in overdose [13]. However, they have been strongly associated with hyponatremia which, in poststroke patients, can worsen outcomes [14, 15, 16, 17, 18, 19, 20].

Hyponatremia, defined as serum sodium concentration < 135 mmol/L has been depicted as a complication of stroke and several studies have reported that hyponatremia of stroke is a predictor of poor prognosis [17, 18, 19, 20]. Identifying the SSRI that is least likely to cause hyponatremia in poststroke patients is crucial due to the high vulnerability of this population to electrolyte imbalances. Even mild, seemingly asymptomatic hyponatremia has also been associated with attention impairment, gait instability, and falls, which could be detrimental to stroke survivors [21]. A literature review comparing hyponatremia risk of individual SSRIs resulted in no conclusive evidence [15]; however, it is interesting to note that a recent study reported the fluvoxamine carries the lowest hyponatremia risk among all SSRIs [22]. It is worth noting that there are currently no published studies comparing the risk of hyponatremia among different SSRIs in poststroke patients. Given the vulnerability of this patient group to develop PSD and hyponatremia, identifying which SSRI carries the lowest hyponatremia risk can inform prescribing practice and enhance patient outcomes.

In Qatar Rehabilitation Institute (QRI), patients diagnosed with stroke are admitted as inpatients to undergo a structured rehabilitation program specifically tailored to stroke survivors. The program aims to enhance recovery, promote independence, and improve the overall health and well‐being of patients, facilitating the journey of poststroke recovery [23]. In QRI, the most commonly prescribed SSRIs for the treatment of PSD are escitalopram and sertraline. This study aimed to determine and compare the incidence and magnitude of hyponatremia and potential risk factors in patients receiving either escitalopram or sertraline for the management of PSD.

## 2 Methods

### 2.1 Ethics Statement

Ethical approval was obtained by the institutional review board (IRB) of the medical research center (MRC) at Hamad Medical Corporation (HMC) in Qatar (MRC‐01‐23‐342). Due to the retrospective nature of the study, informed consent requirement was waved. Anonymity of data was maintained through the study as data were collected and maintained confidentially, with the usage of codes to mask possible identifiers.

### 2.2 Study Design and Settings

This was a retrospective cohort study of all adult patients who received either escitalopram or sertraline for the treatment of PSD during hospitalization at QRI in Doha, Qatar. Patients were identified through an automated report generated electronically by a computerized pharmacy system. The report included patients who were admitted to QRI between February 1, 2019 and February 1, 2024.

### 2.3 Study Participants

Adult patients (≥ 18 years) who were diagnosed with stroke and received rehabilitation care at QRI were targeted in this study. Patients had to receive either sertraline or escitalopram for the treatment of PSD during the study period with documented sodium serum levels at baseline and during follow‐up to be included. Patients who received less than two doses of antidepressant or had no sodium follow‐up (i.e., less than two consecutive readings) were excluded from the study.

### 2.4 Data Collection

Relevant data at baseline and follow‐up were obtained from patients' electronic medical records: sociodemographic (age, sex, and nationality), type of stroke, National Institutes of Health (NIH) stroke scale, dose and duration of antidepressant use, comorbid conditions, and concomitant medications potentially affecting sodium serum levels. The latter includes diuretics (hydrochlorothiazide, indapamide, furosemide, and spironolactone), antiepileptics (levetiracetam, valproate, carbamazepine, pregabalin, gabapentin, topiramate, and lacosamide), antipsychotics (olanzapine, risperidone, and quetiapine), angiotensin‐converting enzyme inhibitors (ACE‐i) (enalapril, ramipril, perindopril, lisinopril, and captopril), angiotensin receptor blockers (ARBs) (valsartan, losartan, and irbesartan), antidiabetics (insulin, gliclazide, glibenclamide, glimepiride, glipizide, and pioglitazone), nonsteroidal anti‐inflammatory drugs (NSAIDs) (ibuprofen, diclofenac, naproxen, etoricoxib, and celecoxib), and proton pump inhibitors (PPIs) (pantoprazole, rabeprazole, lansoprazole, and esomeprazole).

In addition, relevant laboratory data like serum sodium, urea, and serum creatinine concentrations were collected. Baseline sodium serum level was considered as any level within 1 week prior to initiation of antidepressants; all subsequent levels for up to 30 days from initiation of antidepressants were recorded as follow‐up. Hyponatremia was defined as serum sodium level below 135 mmol/L [24]. This was further categorized into mild hyponatremia (130–135 mmol/L), moderate hyponatremia (125–130 mmol/L), or severe hyponatremia (below 125 mmol/L) [24].

### 2.5 Data Analysis

All statistical analyses were performed using the IBM SPSS (Statistical Package for the Social Sciences) version‐22. Both descriptive and inferential statistics were applied for data analysis. Normality testing was performed using Shapiro–Wilk test. Mean ± SD (or median, IQR) and frequency (%) were used to report on numerical and categorical variables, respectively. The level of statistical significance was defined as *p* ≤ 0.05. Pearson's chi‐squared test was used to compare the incidence of hyponatremia between different treatment groups. Student t‐test (or Mann–Whitney test) were used to compare numerical differences across the groups. Multivariate logistic regression was performed to test for the association between the variables of interest and hyponatremia. The factors included in the analysis were age, gender, comorbidities, antidepressants, and the coadministered medications. Odds ratio (OR) with 95% confidence intervals (95% CI) were used to illustrate the correlation between the aforementioned variables and hyponatremia.

## 3 Results

A total of 401 patients met the inclusion criteria and were included in this study. Table 1 describes the baseline characteristics of included patients. Overall, all included patients were male, with a median age of 52 years, and a nationality of mainly South Asian (*n* = 308, 76.8%). Only 14% (*n* = 58) of patients were above 65 years of age. Ischemic strokes were prominent in this cohort (*n* = 260, 64.8%), followed by hemorrhagic strokes (*n* = 113, 38.2%). The most commonly reported comorbidities were hypertension (*n* = 345, 86.0%) and diabetes (217, 54.1%), with 49 (12.2%) patients presenting with either acute or chronic kidney diseases (AKI/CKD). PPIs (*n* = 290, 72.3%), ACE‐i (*n* = 217, 54.1%), antidiabetics (*n* = 145, 36.1%), and antiepileptics (*n* = 129, 32.1%) were the most common concurrent medications received by the studied cohort. Out of the 401 included patients, 175 (43.6%) patients received escitalopram, while 226 (56.3%) patients received sertraline. Median NIH score recorded was 11 and 9 in escitalopram and sertraline groups, respectively (*p* < 0.0001), both of which fill under the moderate risk category. Patients receiving sertraline had significantly higher rates of being diabetics (60.6% vs. 45.7%, *p* = 0.003), and receiving antidiabetic medications (42.0% vs. 28.6%, *p* = 0.006) and antiepileptic medications (40.7% vs. 21.1%, *p* < 0.0001).

**TABLE 1:** Stroke patients' sociodemographic and clinical characteristics.

	Escitalopram		Sertraline		*p* value
Age (median, IQR)	54 (14)		52 (20)		
Gender					
Male	175	100.0%	226	100.0%	
Nationality					
Arab^a^	37	21.1%	41	18.1%	
South Asian	133	76.0%	175	77.4%	
African (non‐Arab)	3	1.7%	5	2.2%	
Others	2	1.1%	5	2.2%	
Stroke type					
Ischemic	106	60.6%	154	68.1%	0.223
Hemorrhagic	57	32.6%	56	24.8%	
Both	12	6.9%	16	7.1%	
NIH score (median, IQR)	11 (5)		9 (5)		**< 0.0001**
Dose (median, IQR)	10 (10)		50 (25)		
Comorbidities					
Hypertension	148	84.6%	197	87.2%	0.471
Diabetes	80	45.7%	137	60.6%	**0.003**
CKD/AKI	20	11.4%	19	8.4%	0.314
Heart failure	3	1.7%	4	1.8%	1
Liver disease/cirrhosis	1	0.6%	0	0.0%	0.436
SIADH	0	0.0%	0	0.0%	Nil
Hypothyroidism	1	0.6%	6	2.7%	0.143
Adrenal insufficiency	1	0.6%	0	0.0%	0.436
Concurrent medications					
Proton pump inhibitors	124	70.9%	166	73.5%	0.575
ACE‐i	97	55.4%	120	53.1%	0.686
Antidiabetics	50	28.6%	95	42.0%	**0.006**
Antiepileptics drugs	37	21.1%	92	40.7%	**< 0.0001**
Diuretics	27	15.4%	25	11.1%	0.231
ARBs	20	11.4%	22	9.7%	0.624
NSAIDs/COX‐2i	10	5.7%	11	4.9%	0.822
Antipsychotics	7	4.0%	6	2.7%	0.572

Abbreviations: ACE‐i, angiotensin‐converting‐enzyme inhibitors; AKI, acute kidney disease; ARB, angiotensin receptor blockers; CKD, chronic kidney disease; COX‐2i, cyclooxygenase‐2 inhibitors; IQR, interquartile range; National Institutes of Health; NSAID, nonsteroidal anti‐inflammatory drugs; SIADH, syndrome of inappropriate antidiuretic hormone secretion.

^a^ Arab countries listed under Arab League classification (i.e., Yemen, United Arab Emirates, Tunisia, Syria, Sudan, Somalia, Saudi Arabia, Qatar, Palestine, Oman, Morocco, Mauritania, Libya, Lebanon, Kuwait, Jordan, Iraq, Egypt, Djibouti, Comoros, Bahrain, Algeria).

**FIGURE 1:**
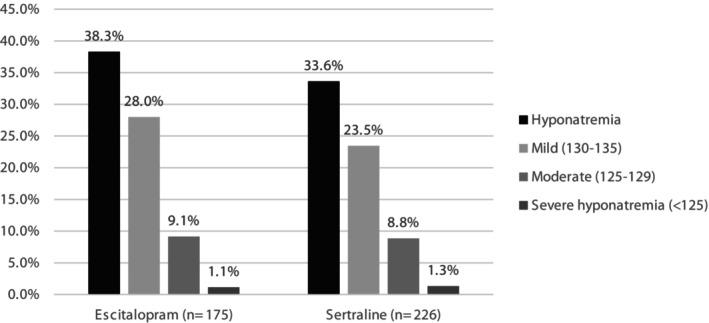
Incidence and magnitude of hyponatremia between escitalopram and sertraline.

Table 2 highlights the changes in laboratory values across the study period. Overall, there was no difference in baseline sodium (138 (4) mmol/L vs. 138 (4) mmol/L), serum creatinine (84 (30) μmol/L vs. 84 (28) μmol/L), or urea (5.9 (3) mmol/L vs. 5.9 (2.8) mmol/L) levels between treatment groups. A total of 143 (36.7%) hyponatremia cases were reported in this cohort, with 67 (38.3%) cases in patients receiving escitalopram and 76 (33.6%) in sertraline group (*p* = 0.256). The median sodium level reported within hyponatremic cases was 132 (3) mmol/L vs. 132 (4) mmol/L in the escitalopram and sertraline groups, respectively, with a median drop of 5 mmol/L in sodium levels from baseline in both groups. Most hyponatremia cases in both groups were of mild nature (73.1% and 69.7% for escitalopram and sertraline, respectively), with only five patients developing severe hyponatremia (3% vs. 3.9% for escitalopram and sertraline, respectively) as shown in Figure 1. Follow‐up serum creatinine and urea levels remained stable during the study period, resulting in no significant change from baseline (Table 2).

**TABLE 2:** Laboratory values obtained from patients' profiles taking either escitalopram or sertraline.

	Escitalopram (*n* = 175)		Sertraline (*n* = 226)		*p* value
Baseline Na (mmol/L)	138 (4)		138 (4)		
Baseline urea (mmol/L)	5.9 (3)		5.9 (2.8)		
Baseline serum creatinine (μmol/L)	84 (30)		84 (28)		
Hyponatremia	67	38.3%	76	33.6%	0.256^a^
Sodium level (median, IQR)	132 (3)		132 (4)		
Drop in Na from baseline (median, IQR)	5 (5)		5 (5.5)		
Mild (130–135 mmol/L)	49	73.1%	53	69.7%	
Moderate (125–129 mmol/L)	16	23.9%	20	26.3%	
Severe hyponatremia (< 125 mmol/L)	2	3.0%	3	3.9%	
Follow‐up serum creatinine (mmol/L)	84 (31)		85 (28)		
Follow‐up urea (mmol/L)	5.6 (2.9)		5.6 (2.9)		

^a^ Chi‐square.

Results from the multivariate logistic regression are summarized in Table 3. Including the predefined variables did not yield a model with significant association (*p* = 0.353). After adjusting for the variables illustrated in Table 3, only the use of ACE‐i resulted in a significant correlation, increasing the odds of hyponatremia by 84% (OR 1.84, 95% CI 1.13–2.99) and suggesting the use of ACE‐i is a predictive factor for hyponatremia development.

**TABLE 3:** Result of multilogistic regression analysis for potential risk factors of hyponatremia.

	OR	*p* value
Hemorrhagic vs ischemic	1.13 (0.67–1.90)	0.64
Both vs. ischemic	1.11 (0.49–2.55)	0.80
Age	1.01 (0.99–1.03)	0.27
Diabetes	1.22 (0.69–2.16)	0.48
AKI/CKD	1.58 (0.75–3.33)	0.23
NIH score	1.04 (0.98–1.10)	0.22
Escitalopram vs sertraline	0.90 (0.57–1.45)	0.68
Diuretics	0.93 (0.49–1.76)	0.83
AEDs	0.95 (0.58–1.54)	0.82
ACE‐i	***1.84 (1.13–2.99)***	***0.01***
ARBs	1.30 (0.59–2.87)	0.52
Antidiabetics	1.31 (0.74–2.31)	0.36
Antipsychotics	0.67 (0.18–2.49)	0.54
NSAIDs	1.09 (0.39–3.02)	0.87
PPI	0.99 (0.60–1.64)	0.97

## 4 Discussion

### 4.1 Statement of Key Findings

To the best of our knowledge, this is the first study to evaluate and compare the incidence, magnitude, and risk factors of hyponatremia in stroke patients receiving treatment with escitalopram or sertraline for PSD. We observed a substantial increase in the incidence of hyponatremia with both escitalopram (38.3%) and sertraline (33.6%) among stroke patients. The median drop in sodium level from baseline was 5 mmol/L in both groups, with most hyponatremia cases being of mild nature (73.1% and 69.7% for escitalopram and sertraline, respectively). Our analysis did not demonstrate a statistically significant difference in the incidence of hyponatremia between both agents. Findings from the multivariate logistic regression did not yield a model with significant association (*p* = 0.353). After adjusting for the predefined variables, only the use of ACE‐i increased the odds of hyponatremia by 84% (OR 1.84, 95% CI 1.13–2.99).

### 4.2 Interpretation and Implications for Future Research and Practice

The incidence of hyponatremia identified in our study is within the range of 9%–40% reported in the literature for SSRIs; however, it is at the higher end of the range [25]. This could be attributed to our population of interest which is poststroke patients who are at increased risk of hyponatremia due to several factors such as the development of the syndrome of inappropriate antidiuretic hormone (SIADH), cerebral salt wasting syndrome (CSWS), and/or dietary sodium restriction as a measure to control hypertension [19, 26]. While multiple studies suggested that the prevalence of hyponatremia during poststroke hospitalization could reach up to 45%; the use of medications was not accounted for in these studies [26, 27, 28, 29]. Therefore, it was not clear whether this finding was due to stroke‐related causes, other causes including drug‐induced hyponatremia, or a combination of both [30]. This study provides the first evidence on the impact of SSRIs' use on the development of hyponatremia among stroke survivors. As a result, clinicians caring for this patient population should exercise caution when prescribing SSRIs and are advised to assess each patient case carefully; accounting for other risk factors that might worsen clinical outcomes.

A plethora of evidence showed that SSRIs are comparable with regard to the incidence of hyponatremia [14, 15, 22, 25]. This is in line with our findings which demonstrated a no statistically significant difference in the incidence of hyponatremia between escitalopram and sertraline. While emerging evidence from a triangulation study suggested that fluvoxamine might be associated with a lower risk of hyponatremia among all SSRIs [22], there remains insufficient volume of research to confirm this conclusion. Additionally, a meta‐analysis comparing the efficacy and tolerability of 21 antidepressant drugs concluded that fluvoxamine is one of the least efficacious drugs for the management of major depressive disorder [31]. However, as none of the published studies focused on the management of PSD, it is recommended that future research incorporates fluvoxamine to provide a better insight into the safety and efficacy profile in poststroke patients. Similarly, a meta‐analysis of 39 studies suggested that mirtazapine could be considered as a treatment option for hyponatremia‐prone patients [32]. Mirtazapine is an atypical tetracyclic antidepressant, and it is not usually the preferred first‐line option; however, a trial of mirtazapine could be considered for patients who experience hyponatremia while taking SSRIs.

Consistent with the results of a Danish register‐based population study that included the general population [14], the majority of hyponatremia cases in our study were of mild nature. This finding should not be taken lightly, as even mild hyponatremia has been linked to attention deficits, gait impairment and increased falls, which could lead to poorer health and rehabilitation outcomes, particularly in stroke survivors enrolled in rehabilitation programs [21, 33]. An expert panel emphasized the importance of identifying and treating mild and minimally symptomatic hyponatremia as it could potentially result in worse health outcomes [34]. Additionally, hyponatremia leads to symptoms ranging from lethargy and anorexia to seizures and coma, which are symptoms that overlap with those of depression [35, 36]. Therefore, it is clinically important to identify SSRI‐induced hyponatremia even if it is mild, and to consider alternative treatment options where possible.

In this study, only the coadministration of ACE‐i significantly increased the incidence of hyponatremia. Several studies and case reports found that the use of ACE‐i alone without any concurrent medications could precipitate hyponatremia through a variety of mechanisms such as stimulating thirst, release of antidiuretic hormone from the hypothalamus, and having a mild diuretic effect, which can be further augmented by the concomitant use of SSRIs [37, 38, 39, 40, 41]. Our study did not identify age as a risk factor; however, this finding should be interpreted with caution as the majority of our participants were younger adults (≤ 65 year‐old). However, this sample is representative of Qatar's residents as the country has a significant population of expatriates, primarily from the Indian subcontinent, who are of working age [42].

### 4.3 Strengths and Weaknesses

This study has several strengths, including a well‐structured design and clear outcome measures. However, a notable limitation is the small sample size. Despite this, the study still provides valuable insights due to its robust methodology and careful monitoring of sodium. While the smaller sample size may reduce the generalizability of the findings, the study's design and execution still contribute meaningfully to our understanding of the relative risks of these medications.

Furthermore, the study has additional strengths related to its focus on a niche patient population—poststroke patients suffering from depression. This focus enhances the study's relevance to a specific and clinically significant group, where the management of depression is complicated by potential risks like hyponatremia. By targeting this population, the study addresses a gap in the literature, as most research on antidepressant‐induced hyponatremia does not specifically examine poststroke patients, who may have different risk profiles due to neurological damage, comorbidities, and concurrent medications. This specificity makes the findings particularly valuable for clinicians managing depression in poststroke patients, providing more tailored insights for this vulnerable group.

Another limitation in this study is the adoption of a definition for hyponatremia with a cutoff value which could introduce potential bias such as overestimating the incidence and possibly hindering the objective evaluation of the drop in sodium level. We have chosen this approach as, in recent years, even mild hyponatremia has been shown to be of clinical importance (due to the subsequent morbidity and mortality), and therefore even mild hyponatremia warrants caution [21, 25, 26]. Moreover, we reported the median drop in sodium levels from baseline to allow for the objective assessment of our findings without the employment of cutoff values or diagnostic criteria. The study was also impacted by the inherent limitations of observational study design such as residual confounding and missing data. Unknown or unmeasured confounders that could influence the risk of hyponatremia could exist such as eating and drinking habits (for instance, psychogenic polydipsia experienced in anxious patients) or inaccurate reporting of SIADH. Additionally, our participants were mainly South Asian and hence generalization of our findings to different populations should be done with caution. Lastly, due to the prescribing practices of physicians in QRI, who preferably prescribe sertraline and escitalopram, it was not possible to include all SSRIs in the study.

## 5 Conclusion

Escitalopram and sertraline were both associated with an increased risk of hyponatremia; however, most documented cases were mild in nature. There was no significant difference between treatment arms regarding the incidence or magnitude of hyponatremia. Caution should be exercised when escitalopram and sertraline are prescribed, particularly in patients with other risk factors that may affect sodium levels such as the concomitant use of ACE‐i.

## Author Contributions

**Lina Naseralallah:** conceptualization, project administration, investigation, data curation, methodology, formal analysis, software, validation, visualization, writing – original draft, writing – review and editing, supervision. **Zahra Noureddine:** conceptualization, project administration, investigation, data curation, methodology, visualization, writing – original draft, writing – review and editing, supervision. **Somaya Koryash:** conceptualization, project administration, investigation, data curation, methodology, formal analysis, software, visualization, writing – original draft.

## Conflicts of Interest

The authors declare no conflicts of interest.

## Data Availability

All data generated or analyzed in this study are included in this article and/or its figures. Further inquiries can be directed to the corresponding author.
